# A novel, hands-free ultrasound patch for continuous monitoring of quantitative Doppler in the carotid artery

**DOI:** 10.1038/s41598-021-87116-y

**Published:** 2021-04-08

**Authors:** Jon-Émile S. Kenny, Chelsea E. Munding, Joseph K. Eibl, Andrew M. Eibl, Bradley F. Long, Aaron Boyes, Jianhua Yin, Pietro Verrecchia, Matthew Parrotta, Ronald Gatzke, Paul A. Magnin, Peter N. Burns, F. Stuart Foster, Christine E. M. Demore

**Affiliations:** 1 Health Sciences North Research Institute and the Northern Ontario School of Medicine, Sudbury, ON Canada; 2 Flosonics Medical, Sudbury, ON Canada; 3 Sunnybrook Research Institute, Sunnybrook Health Sciences Center, Toronto, ON Canada; 4 Department of Medical Biophysics, University of Toronto, Toronto, ON Canada

**Keywords:** Translational research, Ultrasonography, Biomedical engineering

## Abstract

Quantitative Doppler ultrasound of the carotid artery has been proposed as an instantaneous surrogate for monitoring rapid changes in left ventricular output. Tracking immediate changes in the arterial Doppler spectrogram has value in acute care settings such as the emergency department, operating room and critical care units. We report a novel, hands-free, continuous-wave Doppler ultrasound patch that adheres to the neck and tracks Doppler blood flow metrics in the common carotid artery using an automated algorithm. String and blood-mimicking test objects demonstrated that changes in velocity were accurately measured using both manually and automatically traced Doppler velocity waveforms. In a small usability study with 22 volunteer users (17 clinical, 5 lay), all users were able to locate the carotid Doppler signal on a volunteer subject, and, in a subsequent survey, agreed that the device was easy to use. To illustrate potential clinical applications of the device, the Doppler ultrasound patch was used on a healthy volunteer undergoing a passive leg raise (PLR) as well as on a congestive heart failure patient at resting baseline. The wearable carotid Doppler patch holds promise because of its ease-of-use, velocity measurement accuracy, and ability to continuously record Doppler spectrograms over many cardiac and respiratory cycles.

## Introduction

Rapid and reliable hemodynamic assessment is crucial when resuscitating patients in the intensive care unit (ICU)^1^. In circulatory shock, for example, patients often receive intravenous (IV) fluid boluses to increase the amount of blood pumped in a cardiac cycle, the stroke volume (SV), in an effort to normalise cardiac physiology and improve circulation of metabolites. The heart should respond to increased venous return by stretching the cardiac wall at end-diastole, which increases cardiac preload, and increasing left ventricular contraction in order to pump the larger fluid volume through the circulatory system; this is known as the Frank-Starling relationship^2–4^. However, a significant proportion of critically-ill patients do not respond to increased intravenous fluid and cardiac preload, such that stroke volume does not increase. The additional fluids instead contribute to edema and organ injury, which result in poorer patient outcomes^5–7^. Thus, measuring stroke volume change immediately following increased cardiac preload informs the clinician on whether to provide or withhold additional IV fluid^7,8^.

One method of testing the relationship between preload and stroke volume is the passive leg raise (PLR), in which the patient is tilted from a semi-recumbent position to legs raised above the torso. The PLR provides the heart a rapid and reversible bolus of roughly 250 mL of venous blood returning from the abdomen and raised legs^9^. The stroke volume change during PLR therefore indicates whether or not the heart will respond to further intravenous fluid therapy^10–12^. Implicit in the PLR paradigm is the ability to rapidly and reliably measure stroke volume. Non-invasive modalities including bioimpedance, volume clamping, and pulse contour analysis can be used to infer changes in SV and estimate cardiac output^8,13–15^, whereas Doppler ultrasound provides direct assessment. For example, transthoracic pulse-wave (PW) Doppler measurement of blood flow velocity in the left ventricular output tract (LVOT) tracks real-time changes in stroke volume during a PLR^16^.

**Figure 1:**
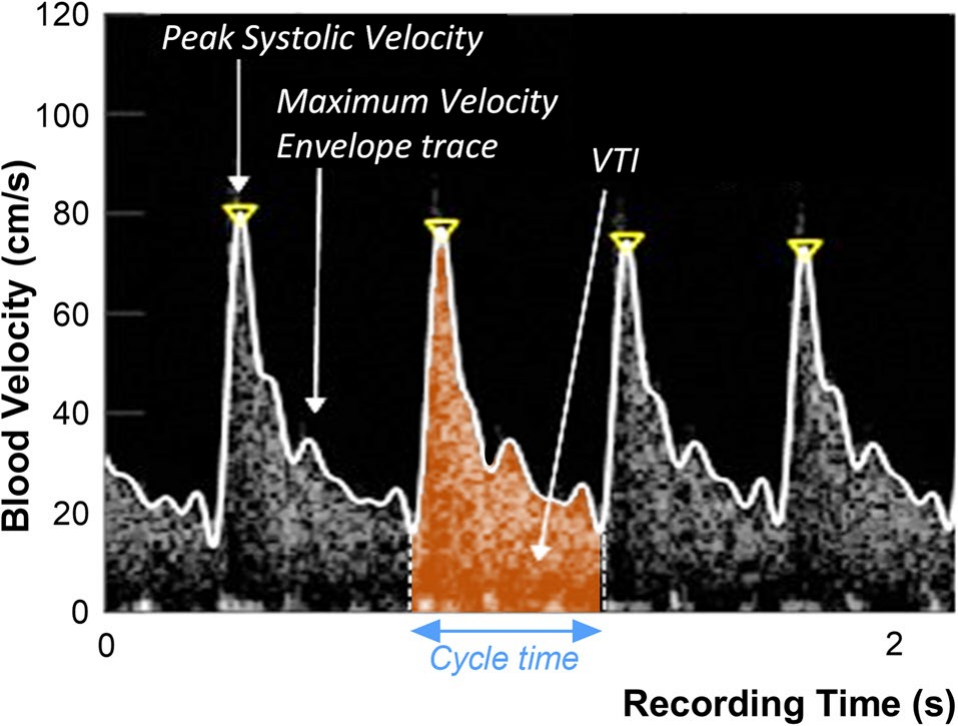
Metrics derived from the spectral Doppler signal for a single cardiac cycle. The velocity time integral (VTI) is the area under the maximum velocity envelope traced from the Doppler spectral waveform for a single cardiac cycle (highlighted in orange) bounded by the cycle time. VTI is also known as the stroke distance and is measured in centimeters.

Using Doppler ultrasonography, stroke volume is measured as the product of the LVOT area and stroke distance, which is the distance the blood travels during a single cardiac cycle. Stroke distance is calculated from the velocity–time integral (VTI), or area under the Doppler spectrogram envelope (Fig. 1)^17–20^. LVOT area is constant, so changes in SV can be evaluated by measuring changes in VTI (i.e., distance)^19,21^. However, transthoracic Doppler is cumbersome to perform repeatedly and imaging the LVOT may be impeded by anatomy and respiratory variation; further, it requires advanced training to obtain and interpret^19,21–23^. Transthoracic and esophageal Doppler have also been used to measure VTI in the descending aorta to infer changes in left ventricular stroke volume, but this also requires cart-based ultrasound imaging equipment and advanced training^24^. In response to these shortcomings, others have successfully used quantitative PW Doppler in the common carotid artery as a surrogate for changing SV to predict the haemodynamic response to PLR over short time periods (i.e., seconds-to-minutes)^25–27^.

**Figure 2:**
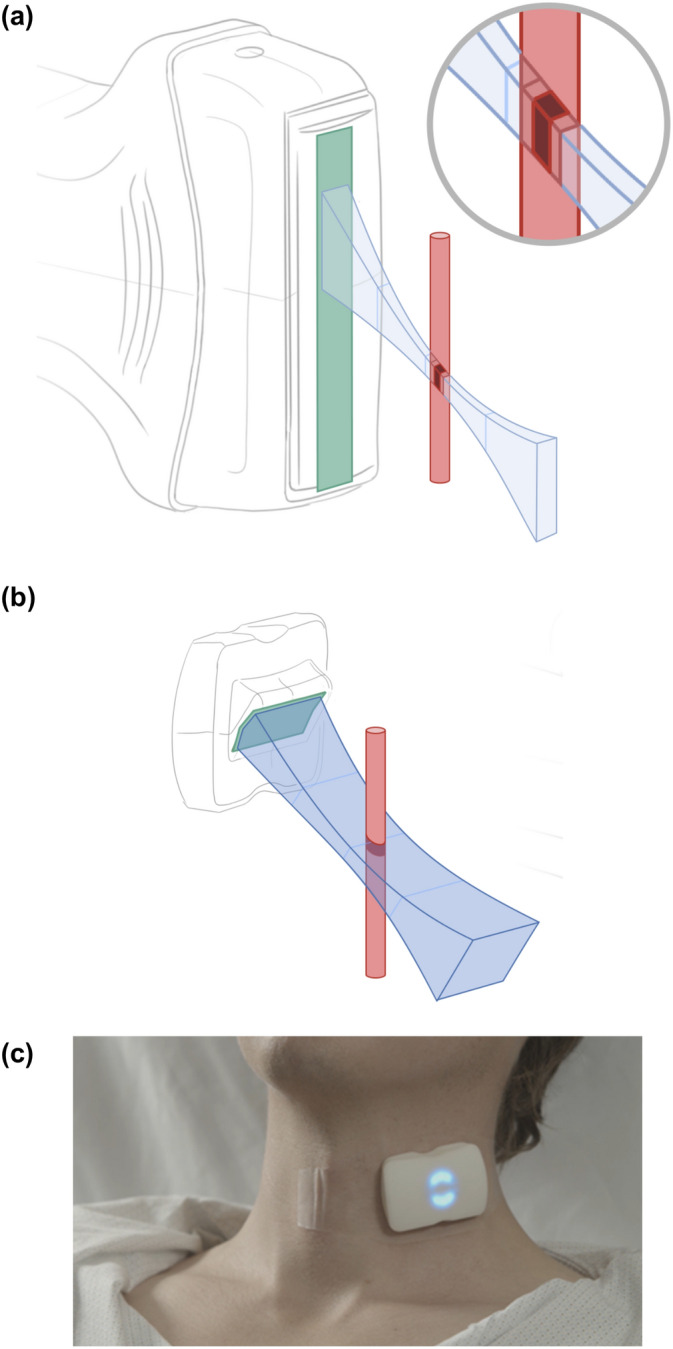
Using Doppler ultrasound to measure blood flow velocity in the carotid artery. (**a**) Using conventional PW Doppler, the handheld transducer is placed on the neck, and the Doppler sample window (dark red) is positioned in the centre of the artery. The Doppler angle cursor (black dashed line, see inset) is then aligned with the direction of blood flow to obtain an accurate velocity measurement. (**b**) With a broad-beam CW Doppler patch, the device is placed on the neck with the wide beam intersecting the cross-section of the carotid at approximately 60°. Doppler signals are simultaneously acquired from the entire beam area. (**c**) An adhesive strip secures the CW Doppler patch on the neck to maintain its position and angle over the course of repeated measurements.

Nevertheless, PW Doppler remains challenging because it is a skill-dependent, ‘hands-on’ technique. It can be difficult to maintain the probe position consistently over the vessel of interest while acquiring Doppler spectrogram measurements at baseline and during the PLR, introducing variability^28,29^. The operator must manually orient the ultrasound image plane with the vessel axis, place the PW Doppler sample window at the center of the vessel (e.g., Fig. 2a), and align the angle cursor with the longitudinal axis of the vessel to determine the Doppler angle (*θ*) between beam axis and flow direction. Centering the sample window and ensuring uniform insonation is important for accurate estimation of flow velocities, particularly for vessels that have a blunted paraboloid flow profile, such as the common carotid artery^30,31^. The blood velocity, *v*, is calculated using the Doppler equation:

1 $$v = \frac{c\Delta f}{{2f_{0} \cos \theta }},$$

where *c* is the speed of sound in the tissue, *f*_*0*_ and *Δf* are, respectively, the frequency of the transmitted ultrasound wave and the Doppler-shift measured by the imaging system^32^. The range of *Δf* included in the acquired spectrogram reflects the range of velocities detected within the ultrasound beam. The angular alignment is therefore critical for accurate velocity measurement: for a nominal Doppler angle of 60°, a 5° error in alignment results in approximately 15% error in velocity^28^. Further, as it is the change in measured VTI that infers changing SV, alignment variability between measurements (e.g., due to patient or probe movement when the patient is reclined during a PLR maneuver) will produce errors not only in the absolute velocity measurements, but compounded error in the relative change in VTI.

Considering these factors, we have developed a novel, hands-free, continuous wave (CW) Doppler patch to easily and continuously monitor changes in blood flow velocities in the common carotid artery. The Doppler patch generates an ultrasound beam significantly wider than the carotid artery to facilitate easy placement of the probe over the artery and uniform insonation (Fig. 2b). Further, the CW Doppler patch adheres to the neck (Fig. 2c), making the sensor hands-free. Importantly, this design allows for consistent positioning and alignment between the beam and the artery throughout a PLR manoeuvre, limiting changes to the Doppler angle (Eq. 1) so that clinically relevant indices such as VTI can be acquired continuously and compared quantitatively. As with PW Doppler, an error in the angle from the nominal value will result in an erroneous absolute estimate of velocity; however, by maintaining the angle of insonation the measured change in velocity will be correct, notwithstanding the deviation from the nominal angle.

In this paper, we present the design of an adhesive, hands-free CW Doppler ultrasound patch for detecting blood flow quickly and easily in the common carotid. The sensor is combined with an automated algorithm to trace the continuously-recorded velocity waveform and calculate haemodynamic indices such as VTI. The accuracy of the Doppler patch in detecting a change in flow velocity is evaluated in vitro using moving string and blood mimicking flow phantoms. The ease-of-use of the device is demonstrated in a small usability study. Finally, we demonstrate the feasibility of using the device in vivo by continuously capturing and recording pulsatile flow in a resting patient, as well as measuring VTI during a PLR manoeuvre in a healthy volunteer.

### Hands-free CW Doppler system design

**Figure 3:**
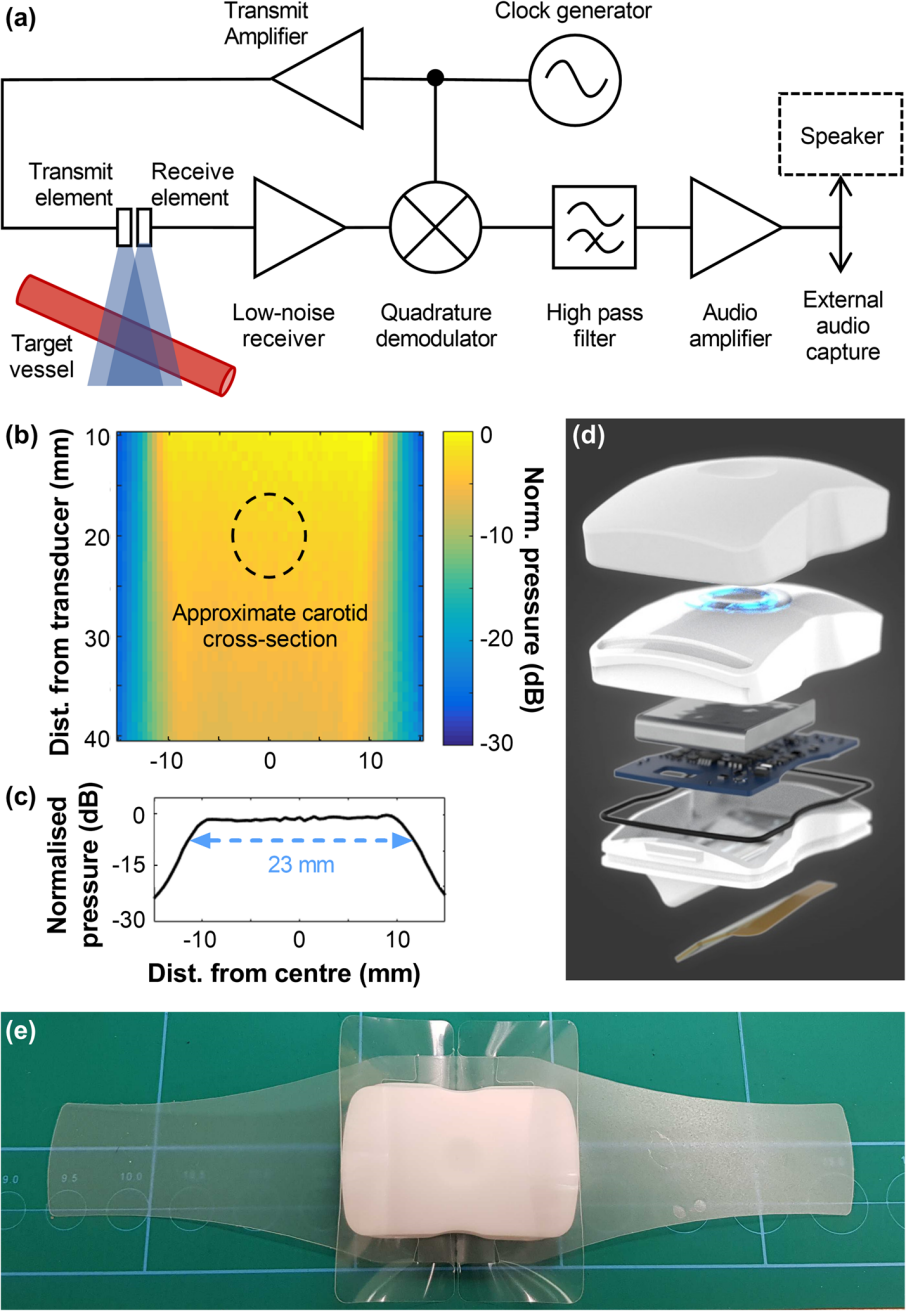
The CW Doppler patch. (**a**) System diagram showing the transmit and receive electronics paths. (**b**) Pressure map of the wide transmitted ultrasound beam measured in the lateral plane with a hydrophone, normalized to the peak pressure and log-scaled; with an outline of the approximate cross section of a 7-mm-diameter carotid artery superimposed on the beam (black dashed line). (**c**) Transmit beam pressure profile, log-scaled, at distance of 1 cm from the transducer showing the uniformity of the pressure amplitude (± 0.7 dB) over the active region. (**d**) Exploded view of CW Doppler patch components, including (from bottom) transducer module mounted in angled wedge, water-resistant gasket, PCB for Doppler processing and wireless communication, rechargeable battery, case with indicator LEDs, and silicone sleeve. Image by Kyle Fredricks/Flosonics Medical. (**e**) Wireless configuration of device, on a 2″ grid for scale, with translucent adhesive strips on each side and clear tabs, visible around the patch, to remove liner of adhesive.

The carotid CW ultrasound patch (Flosonics Medical, Sudbury, Canada) is a wearable, non-invasive device (Fig. 3) that uses two continuous-wave 4 MHz ultrasound transducers; one transducer transmits sound continuously, while the other receives echoes continuously. The frequency of the received echoes, including those backscattered by blood, is compared to that of the transmitted beam; frequency shifts due to the Doppler effect are resolved by the receiver electronics (Fig. 3a). The change in frequency—the Doppler shift—is proportional to the velocity of motion towards or away from the transducer and can be measured by analysing the spectrum of the Doppler signal. In this way, moving blood anywhere within the overlapping volume of the two beams can be detected and the component of its velocity in the direction of the transducer characterised. The Doppler spectrum is calculated at 12.8 ms intervals, and combined with Eq. (1), provides a temporal measurement of the range of detected velocities.

Both transducers in the CW ultrasound patch have a wide aperture (approximately 23 mm) to generate a uniform broad beam (Fig. 3b,c), enabling easy signal acquisition and uniform insonation of a target vessel. The beam width is roughly 3–4 times the width of the carotid artery. This facilitates the correct placement and minimizes the need for repositioning to capture the flow signal. It is worth noting that this ease-of-use comes at the cost of a lower Doppler signal-to-noise ratio, since the device is receiving ultrasound echoes from the entire insonated field, of which only a small portion contains moving blood that produces the Doppler-shifted echoes. The relatively superficial location of the carotid, however, makes this trade-off acceptable for the present application. The broad, unfocused beam also distributes the ultrasound energy and minimizes the risk of tissue heating.

The probe housing contains the transmit and receive transducers and, in a fully wireless configuration, the Doppler processing electronics and battery (Fig. 3d). The housing is wedge shaped so that the ultrasound beams intersect the short-axis of the artery with an angle of approximately 60 degrees relative to the direction of flow. Ultrasound gel is used as an acoustic couplant between the outer face of the wedge and the skin. As shown in Fig. 3e, medical adhesive straps (3 M, Minneapolis) fasten the device to the subject’s neck, fixing the orientation and position relative to the skin above the carotid artery. The adhesive can be worn in excess of 24 h, while the battery-powered device can acquire velocity measurements continuously for more than 180 min, or intermittently for longer monitoring durations.

The device electronics pair wirelessly with a tablet-based application for data recording and Doppler spectrum analysis, using Eq. (1) to calculate blood flow velocity and assuming a 60° insonation angle. An audible Doppler signal is produced by the system to facilitate placement, then muted for subsequent measurements while Doppler velocity waveforms are continuously recorded. An example the spectrogram and maximum velocity trace for a volunteer subject is shown in Fig. 1. Maximum flow velocities are estimated with an automated tracing algorithm based on the approach described by Steinman et al.^33^, and subsequently used to calculate the VTI and pulse rate. In the present studies, the waveform tracing was performed after data acquisition had been completed. However, this algorithm can be implemented for real-time segmentation and calculation of VTI so that blood flow metrics can be displayed, together with a visual trace of the carotid blood velocity waveforms.

## Results

### Doppler characterization in vitro

**Figure 4:**
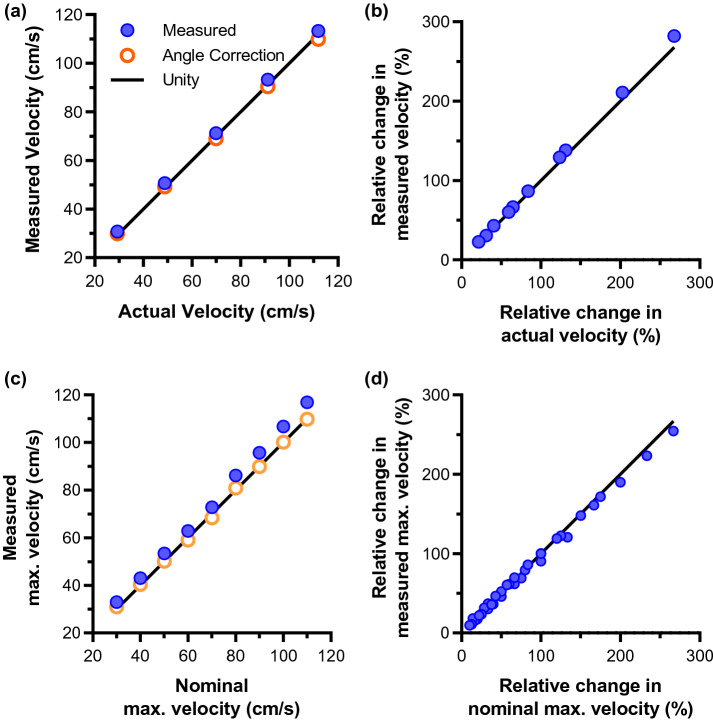
In vitro validation results of CW patch velocity measurement, showing good tracking of absolute and relative velocities. (**a**) Absolute velocity measured by CW patch on a string phantom. A 1° angle correction improves the agreement between measured and actual velocities. (**b**) Comparison between measured relative change in velocity and actual relative change on a string phantom. (**c**) Comparison of absolute measured maximum velocity and nominal maximum velocity on a flow phantom. A 2° angle correction improves the agreement. (**d**) Good agreement between measured and nominal maximum velocity in a flow phantom.

Moving string test objects and flow phantoms with blood mimicking fluid were used to test the CW Doppler patch and evaluate measurement accuracy of both absolute and relative change in velocity. Recorded measurements of the moving string absolute velocity showed excellent agreement with actual string velocities (Fig. 4a), with a maximum error (at 110 cm/s) and mean error of 5.1% and 3.9%, respectively. This error is reduced to 2.9% (at 30 cm/s) and 1.3% if a corrected insolation angle of 61° is used in Eq. (1). Although the validation of absolute velocity accuracy is helpful as a demonstration of the device performance, for the target application of measuring changes in VTI it is the change in velocity that is important. It should be noted that the purpose of attempting the angle correction was simply to show that the majority of the error on the absolute velocity can be accounted for by a deviation from the nominal angle. When measuring a relative change in velocity, the angle can be ignored as long as the same angle is maintained for both measurements. To demonstrate the ability to measure velocity change, each permutation of relative change in velocity between the five velocity settings was compared using the nominal insonation angle of 60° (Fig. 4b). In general, relative change in velocity was estimated well. The poorest measure, by relative error, among these permutations was a 40.4% measured change for an actual change of 43.0% (6.4% relative error). On average, a difference of 4.5% between the actual and measured change in velocity was found, or a relative percentage error of 4.1%.

Initial velocity measurements using a constant flow phantom showed somewhat poorer agreement between the measured and expected maximum absolute velocities (Fig. 4c), with maximum error (at 30 cm/s) of 10% and mean error of 6.7%. Using a corrected insonation angle of 62°, however, reduced the maximum error (at 30 cm/s) and mean error to 3.3% and 1.1%, respectively. Agreement with the relative velocity change (Fig. 4d) was also somewhat poorer than with the string phantom, with the poorest measurement being a 14.3% measured change for an 18.4% nominal change (a relative percentage error of 28.8%). The average difference and relative error, however, were both low: 3.5% and 6.0%, respectively.

#### Ease-of-use study

A small usability study evaluated whether 22 participants as new users (17 with clinical background; 5 lay) were able to deploy the CW Doppler patch on one of three healthy volunteer subjects, as well as their perception of the ease-of-use. All were successfully able to place the device over the subject’s carotid artery and obtain clear, audible and recognizable Doppler signals. In a survey of their first experience using the device, all users “strongly agreed” (n = 19) or “agreed” (n = 3) that the device was easy to use. Furthermore, all users “strongly agreed” or “agreed” that the device was easy to place over the carotid artery (n = 19, n = 3) and that the Doppler sound was clearly audible (n = 21, n = 1). No unforeseen risks were identified during the study, but some minor changes to the labelling and adhesive strap design were identified to enhance the ease-of-use. The subjects did not note any discomfort due to the wedge design, temperature increase of the transducer, or any discomfort from the gel.

#### In vivo measurement of carotid VTI

**Figure 5:**
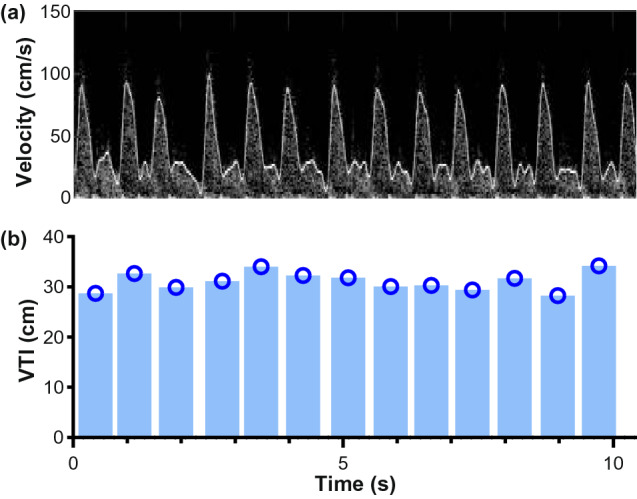
(**a**) Continuous carotid Doppler waveform monitoring with automatically traced maximum velocity envelope (white line). (**b**) Beat-to-beat quantification of carotid velocity time integral (VTI).

We tested continuous real-time spectral Doppler signal acquisition using CW Doppler patch in a patient with congestive heart failure during quiet respiration as proof-of-concept demonstration of the system capabilities. The hands-free device easily adhered to the neck, captured the audible Doppler shift and continuously captured Doppler spectra from the common carotid artery. As shown in Fig. 5, the automated maximum velocity tracing algorithm qualitatively tracked the Doppler spectrograms, and VTI was calculated for each beat. Beat-to-beat variability, which is expected for this patient, can be observed in the spectrogram, the automated trace and the calculated VTI values.

**Figure 6:**
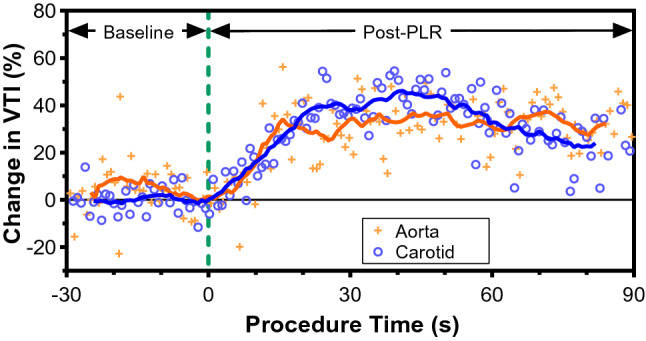
Time course of velocity time integral (VTI) following a passive leg raise (PLR) intervention to modulate cardiac preload in a healthy volunteer. The leg raise was initiated at t = 0 s (vertical dashed-line). VTI is calculated from Doppler waveforms acquired simultaneously from the carotid using the CW Doppler Patch (blue, open circles) and the descending aorta using PW Doppler (orange, crosses) and normalized to the mean VTI between  − 10 and 0 s of baseline. Solid lines are moving average of 12 beats.

We assessed the ability of the CW Doppler patch to detect changes in common carotid flow velocity and stroke distance during a passive leg raise maneuver in a healthy volunteer. Measurements of aortic spectral Doppler were acquired simultaneously with PW Doppler via the suprasternal notch using a cart-based ultrasound imaging system. Figure 6 shows the time course of normalised aortic and common carotid VTI (i.e., stroke distance in centimeters), for the PLR manoeuvre, beginning with 30 s of baseline measurements in the semi-recumbent position. The moving average curves, using 12 points, are shown to elucidate the temporally-evolving trends. Following initiation of the PLR, there is augmentation of both aortic and carotid VTI for the duration of the test. At 30 s post-PLR, both the aortic and carotid VTI have increased by 30 to 40% compared to baseline, indicating a positive response to cardiac preload for this healthy subject.

## Discussion

A novel CW Doppler patch was developed to quickly and reliably capture quantitative Doppler flow velocity traces of the common carotid artery in real-time. The Doppler patch facilitates placement due to its form factor, with long elements that generate a broad beam. This design was chosen to uniformly insonate the entire vessel cross-section to allow quick and intuitive positioning over the carotid artery. This ease-of-use was demonstrated in a small usability study, which confirmed that all volunteer users, both those with and without clinical training, were able to obtain an audible Doppler signal and felt that the device was easy to use. The ease of placement is well-suited to a fast-paced clinical environment, especially when compared with the time and expertise needed to setup a conventional cart-based ultrasound scanner and obtain an accurate Doppler signal.

The accuracy of the CW Doppler patch in identifying changes in target velocity was demonstrated in string and flow phantom experiments. Excellent absolute measurement accuracy, with the variation of expected velocities, was obtained for both string and laminar flow phantoms when a small (≤ 2°) correction in the insonation angle was implemented. Although the evaluation of absolute velocity accuracy is useful for validating the device and system performance against specification, it is the relative change in blood flow velocity that is important in functional hemodynamic monitoring. This relative change in velocity is unaffected by the angle of the ultrasound beam relative to the target, provided the angle is maintained throughout each subsequent measurement so that *Δf* is the only variable in Eq. (1). Using the nominal 60° angle of insonation to calculate velocity, relative changes in measured velocity closely match expected values for the string and laminar flow phantoms, with mean error ≤ 6% across the range of target velocity pairings. The CW Doppler patch is held in place on a subject’s neck by adhesive strips to maintain a stable insonation angle. This design facilitates repeated measurements and removes a potential source of error in calculating changes in velocity since the alignment between the beam and artery is maintained.

As demonstrated in the volunteer during PLR and quiet respiration, the Doppler ultrasound patch was able to qualitatively and quantitively follow pulsatile flow continuously in the common carotid artery. The healthy volunteer had an approximately 30–40% rise in aortic VTI during the PLR, which is considered clinically significant (> 10%)^34^ and indicates the heart increased stroke volume in response to increased cardiac preload. This increase in VTI was also detected by Doppler patch at the common carotid artery, with a similar rise within 10–15 s of initiating the PLR.

The design and function of the CW patch are clinically-relevant for many reasons. A well-established method for predicting the response to intravenous volume expansion is the PLR with simultaneous assessment of VTI in the left ventricular output tract (LVOT)^16^. Because transthoracic quantitative Doppler may be cumbersome to perform repeatedly at the bedside, the use of a peripheral artery to track SV change is a desirable surrogate. Indeed, previous investigations have found that carotid Doppler is an acceptable approach to functional hemodynamic monitoring^25,26^. We have previously shown in healthy volunteers performing squat maneuvers that a 10% rise in SV corresponds to a 15% VTI threshold^35^. A rise was also observed during PLR in a healthy volunteer in the present study. However, not all clinical studies have replicated these findings^36^. One contributing factor to these discrepancies may be the small number of beats sampled (e.g., 1–3) used to calculate blood flow metrics coupled with normal, inherent VTI variation introduced by the respiratory cycle^37–39^. The hands-free CW Doppler patch is well-suited to overcome this potential source of variability by acquiring continuous measurements over many beats^20^. The preclinical phantom, healthy volunteer and congestive heart failure patient data shown here demonstrate that the device can accurately measure changes in flow velocities as well as continuously track changes in carotid VTI during a PLR maneuver.

A number of important limitations of our study must be acknowledged. First, we measured stroke distance (VTI) and not absolute flow rate. We opted to measure VTI rather than volume flow because the latter would require calculation of vascular area derived from diameter measurement, and any error in the linear measurement of diameter is amplified to the second power^32^. Because we studied a healthy subject with normal blood pressure, we reasoned that changes in vascular diameter would be minimal. This is likely to be less true in critically-ill patients where measurement of vascular diameter does have implications for assessing fluid responsiveness in the descending aorta^40^. Further, the utility of the device will need to be characterized in abnormal anatomy likely to be encountered in a clinical setting, such as stenotic and tortuous vessels. Nevertheless, because the qualitative changes in VTI during PLR matched what is expected for volume flow, we feel that VTI was a viable surrogate in the healthy volunteers.

In conclusion, we describe the application of a novel hands-free continuous wave Doppler ultrasound patch fixed in place over the common carotid artery to continually measure blood flow velocities. An automated algorithm has been implemented to automatically segment the Doppler waveforms and calculate blood flow metrics. The small usability study demonstrates that both lay and clinical users can readily position the CW patch to record Doppler waveforms, while the string and flow phantom studies demonstrates accuracy in identifying changes in velocities. In a proof-of-concept in vivo study, the CW Doppler patch was able to continuously record and track instantaneous changes in carotid VTI during a PLR in a healthy volunteer and during quiet respiration in a patient with congestive heart failure. This Doppler patch may be useful for functional hemodynamic monitoring in the emergency department, operating room, and intensive care unit; further investigation in these settings is planned.

## Methods

### In vitro velocity measurement validation

A series of in vitro experiments was performed to ascertain the accuracy of measuring changes in flow velocities with the CW Doppler patch. Absolute velocity accuracy was established using a motorized string in a water tank: a loop of nylon string was continually moved through a system of pulleys, with a straight segment of the loop passing through the ultrasound beam in the elevational direction. The string velocity was set to 30, 50, 70, 90 and 110 cm/s via the motor controller. For each nominal velocity setting, the actual string velocity was calculated by dividing the known string length by the time interval for the joining knot to pass through the beam. A custom jig held the transducer in position with an angle of approximately 60° relative to the string. For each velocity setting, a 2-s-long spectrogram was acquired and averaged over time. The Doppler shift used to calculate string velocity (Eq. 1) was determined by finding the peak amplitude of a Gaussian curve fitted to the averaged power spectrum.

To account for deviation from the nominal θ = 60° angle between the ultrasound beam and moving target due to manual placement of the probe, which results in errors in the measured absolute velocities (Eq. 1), the angle used in the velocity calculation was modified by 1° increments. The corrected angle is that which yields the smallest mean error in absolute velocity measurement.

To assess the performance of the device in measuring a change in velocity, each permutation of relative change in velocity, *v*_*rel%*_, was calculated as:

2 $$v_{rel\% } = \frac{{v_{j} - v_{i} }}{{v_{i} }} \times 100$$

where *i* and *j* represent the velocity settings, and *v*_*i*_ < *v*_*j*_.

The performance of the CW Doppler patch in measuring maximum velocity in a laminar flow profile was assessed using a constant flow phantom setup comprising a programmable pump, tissue mimicking Doppler flow phantom with a 6-mm-diameter channel (Doppler Flow Pump Model 769 and Peripheral Vascular Doppler Flow Phantom Model ATS 524, CIRS Inc., Norfolk, VA, USA), and blood-mimicking fluid (BMF-US, Shelley Medical Imaging Technologies, London, ON, Canada). A custom jig was used to hold the probe approximately 1.5 cm above the flow channel with an angle of approximately 60° between the axial direction of the ultrasound beam and the direction of flow in the channel. Ultrasound gel was used as a couplant between the device and the phantom surface. Spectrograms were recorded for programmed maximum flow velocities between 30 and 110 cm/s with intervals of 10 cm/s. The automatic tracing algorithm was used to calculate the maximum velocity trace of spectral Doppler signals received by the CW Doppler patch.

### Usability study

A small usability study was conducted to identify potential difficulties or hazards that might arise among new users. The study involved 22 volunteer users (5 physicians, 11 nurses, 1 technician, 5 lay users) using the CW Doppler patch for the first time on one of three volunteers. The users were given a brief demonstration of deploying the CW Doppler patch, then observed as the user attempted to position the device over the subject’s carotid artery, obtain an audible Doppler signal then fix the adhesive strap in place. Audible Doppler was chosen as the usability study endpoint because audible Doppler assessments are common in nursing training. Users were given a brief survey to assess their experience with the device. They were asked to rate their agreement with a scale from 1 to 5, with 1 indicating “strongly disagree” and 5 indicating “strongly agree”. The relevant survey statements were (1) “I found the device easy to use”, (2) “I found it easy to maneuver the device over to the carotid artery”, and (3) “I was able to hear the Doppler sound clearly”. Other statements on the survey referred to details unrelated to the essential functionality of the device, so are omitted here.

### In vivo proof-of-concept

We recruited a healthy adult volunteer (35-year-old female, 37 cm neck circumference, BMI of 25.1) with no known cardiovascular history and on no regular cardiovascular medications. We also recruited one stable patient on the general medical floor admitted for congestive heart failure (94-year-old male, 44 cm neck circumference, BMI of 24.8). The study was approved by the Research Ethics Board of Health Sciences North, Sudbury, Canada and the study conducted in the associated Simulation Lab in accordance with all institutional regulations and guidelines. Written informed consent was obtained from the subjects.

Real-time carotid Doppler waveforms were recorded from the congestive heart failure patient using the CW Doppler patch during quiet respiration. The subject was in the semi-Fowler, or semi-recumbent position (torso at 45° to horizontal) on a standard gurney. The CW Doppler patch was placed, by palpation over the participant’s left common carotid artery and below the angle of the left jaw (as in Fig. 2c) in order to sample flow below the bifurcation. The device was secured once a sufficiently strong and reproducible spectrogram signal was audible, and visualized on the tablet-based recording application. No other adjustment of the hands-free device was made during the test. The spectral CW Doppler signals were recorded continuously until the end of the test, totaling several minutes. The maximum velocity envelope of the carotid CW Doppler waveforms recorded was extracted with the automated tracing algorithm. VTI was calculated as the area under the curve for each beat detected with the algorithm.

For the healthy volunteer, Doppler signals were acquired simultaneously in the carotid with the hands-free device and in the descending aorta using PW Doppler from a cart-based ultrasound imaging system (Xario, Toshiba, Tokyo, Japan) while a PLR was performed, as described below. A cabled version of the CW Doppler patch, in which the transducer assembly is connected by coaxial cables to an external electronics box, was used for the proof-of concept demonstration to facilitate synchronous spectral Doppler signal capture from both the prototype device and the ultrasound imaging system. The external Doppler audio output from both the CW Doppler patch and the cart-based imaging system were fed into a two-channel audio recorder (Roland Corporation, Los Angeles, CA) and visualised using an open access audio-recording program (Audacity, https://www.audacityteam.org). The CW Doppler audio output fed into the Roland audio recorder is the same signal that is transferred to the tablet-based recording application by the wireless version of the device.

The healthy volunteer was studied fasting in the morning. The PW Doppler measurements in the descending aorta were acquired by a trained sonographer blinded to the data acquired simultaneously from the hands-free Doppler ultrasound patch. A phased array cardiac probe (7.5 MHz) was positioned in the suprasternal notch to obtain a view of the descending aorta, confirmed by identification of the left subclavian artery and pulsatile flow away from the probe, as previously described^24^. The sample window was positioned mid-vessel with insonation angle of 0°, since flow was directly away from the probe, and sample window length of 4 mm. During the PLR, the sonographer manually adjusted the probe as required to keep the sample volume at the center of the vessel and maintain angle of insonation. The PLR manoeuvre was performed, beginning with 30 s resting baseline in the semi-Fowler position, followed by lowering of the torso to horizontal with the legs raised to 45 degrees for 90 s. Care was taken to minimize subject contact to ensure that the maneuver was fully passive.

The carotid CW Doppler and aortic PW Doppler spectral waveforms recorded from the healthy volunteer were exported as images from the Audacity software then imported into digital tracing software WebPlotDigitizer (https://apps.autoeris.io/wpd/). Data points outlining the maximum velocity envelope were manually selected. Hand-tracing of both the aortic and carotid Doppler waveforms was performed by one of the authors (JESK). Hand-tracing was employed because automated tracing software was not available for the aortic spectrogram. In order to readily compare blood velocity changes in the aorta to the carotid, which have different absolute VTIs, the VTI was normalized to the mean VTI for each vessel during resting baseline (time window: -10 to 0 s). The relative change in VTI was calculated using Eq. (3).

3 $$VTI_{\% \,change} = \frac{{VTI_{j} - VTI_{i} }}{{VTI_{i} }} \times 100\% ,$$

where the *i* and *j* subscripts represent the pre- and post-intervention VTI, respectively.

## Data Availability

The data used in this study can be made available on request.
